# School composition, family poverty and child behaviour

**DOI:** 10.1007/s00127-016-1206-7

**Published:** 2016-04-08

**Authors:** Eirini Flouri, Emily Midouhas

**Affiliations:** Department of Psychology and Human Development, UCL Institute of Education, University College London, 25 Woburn Square, London, WC1H 0AA UK

**Keywords:** Child behaviour, MCS, Millennium Cohort Study, Poverty, School composition

## Abstract

**Purpose:**

There is little research on the role of school composition in young children’s behaviour. School composition effects may be particularly important for children in disadvantaged circumstances, such as those growing up in poverty. We explored the role of school academic and socio-economic composition in internalising problems, externalising problems and prosocial behaviour at age 7 years, and tested if it moderates the effect of family poverty on these outcomes.

**Methods:**

We used data from 7225 7-year-olds of the Millennium Cohort Study who attended state primary schools in England and for whom we had information on these outcomes. In multiple membership models, we allowed for clustering of children in schools and moves between schools since the beginning of school, at age 5. Our school academic and socio-economic composition variables were school-level achievement and % of pupils eligible for free school-meals, respectively. Poverty (family income below the poverty line) was measured in all sweeps until age 7. We explored the roles of both timing and duration of poverty.

**Results:**

The effects of poverty were strong and robust to adjustment. School socio-economic composition was associated with individual children’s internalising and externalising problems, even in adjusted models. School composition did not interact with poverty to predict any of the outcomes.

**Conclusions:**

Neither the academic nor the socio-economic composition of the school moderated the effect of family poverty on children’s behaviour in primary school. However, children attending schools with more disadvantaged socio-economic intakes had more internalising and externalising problems than their counterparts.

## Introduction

Family poverty is strongly associated with children’s emotional (internalising) and behavioural (externalising) problems [1–7]. The pathways linking poverty and child emotional/behavioural problems are parental ill mental health [8], weakening of family relationships, disengaged and harsh parenting practices, and/or lack of resources to purchase services and materials that benefit child well-being [9]. However, some children manage to escape the consequences of poverty [10, 11], perhaps due to individual characteristics, family qualities or environmental influences working together to forge resilience through a dynamic process [12]. An environmental factor related to children’s emotional/behavioural resilience to poverty may be school composition (or ‘mix’). This study was carried out to test this.

School-composition effects refer to the collective, rather than the individual, influence of pupil characteristics, and composition is the aggregation (at the school-level) of pupils’ characteristics, including demographic, socio-economic or academic/intellectual [13–16]. In essence, school-composition effects capture the influence of pupils’ peer groups. Some research has supported the role of the socio-economic [17] and academic [18] composition of the school in predicting individual academic performance. There is also recent evidence for the role of such school ‘effects’ in children’s health outcomes [19] as well as suggestions for gender differences in such effects in adolescence [20]. There is little research, however, on the role of school composition in explaining individual pupils’ differences in psychological outcomes. This limited evidence shows that the socio-economic rather than the academic intake of the student body influences the emotional/behavioural outcomes of individual children, and that effects are small [21–26].

Although school composition may have a small impact on pupils’ behaviour, it may be more important for the behaviour of pupils from socio-economically disadvantaged backgrounds. Schools can substantially halt or even reverse the effect of family poverty on children’s academic or cognitive outcomes, especially if interventions towards and investments in disadvantaged children are made early [27]. Yet, research has not explored the role of school composition in reducing the effect of poverty on children’s emotional/behavioural problems. Theory of contextual effects on individual outcomes suggests two reasons why attending a school with a privileged socio-economic or academic intake may be particularly beneficial for the emotional/behavioural outcomes of disadvantaged children. One is because of positive peer contagion, namely the upward-levelling norms of high-achieving or well-behaving peers [28, 29]. A second way is through institutional characteristics that may relate to favourable pupil characteristics, including higher parental involvement in schooling, higher-quality teachers, more effective management processes within schools and a more rigorous curriculum [30]. These characteristics may compensate for a more chaotic, less organised home environment, and one where the child receives less social support and less responsive parenting, all of which are more common in poor families [31] and strongly associated with children’s emotional/behavioural problems [32].

Nevertheless, there is other theory and research suggesting that school socio-economic composition effects may be different for advantaged and disadvantaged children, but in the opposite direction, as poor children in such schools may experience feelings of social inferiority [33], in turn associated negatively with achievement and mental health. Simply put, attending a school with a higher socio-economic status (SES) intake may have a detrimental rather than a positive effect for children in poverty due to relative deprivation mechanisms [14]. Although not consistently [34], research has certainly shown that students from relatively advantaged backgrounds tend to derive greater educational benefits from attending high-SES schools [35, 36], suggesting that high-SES schools perpetuate social reproduction [37]. Although we are mindful of these findings and the theory to support them, we think that any added advantage of being high-SES in a high-SES school may be age dependent. School ‘choice’ (and therefore the role of schools in perpetuating social reproduction) may become more important for families as children grow older because of the predictive role of performance later in school for future outcomes. The role of school academic composition (usually measured as school-average achievement) in individual children’s outcomes has attracted more research interest but, again, findings are mixed. Some studies find negative effects [23], in line with predictions from the theory of relative deprivation, others positive effects, and few non-linear effects, in line with other evidence that the effect of student composition changes as it moves toward a potential tipping point [18].

### The present study

To our knowledge, this is the first study to investigate whether school composition can moderate the association between family poverty and primary school children’s behaviour. Our study used large-scale longitudinal data from the UK’s Millennium Cohort Study (MCS) and had three aims:

1. To model the relationship between family poverty across early-to-middle childhood (ages 9 months to 7 years), in terms of both the duration of exposure and its timing, and child behaviour (measured as internalising and externalising problems and prosocial behaviour at age 7).

We hypothesised that, even after accounting for individual and family characteristics, family poverty would be associated with children’s behaviour, given prior research demonstrating this relationship.

2. To explore the role of school composition—academic and socio-economic—in both predicting child behaviour and moderating the effects of poverty on child behaviour.

We expected to find that attending a high-achieving school or a school with a socially-privileged intake would be related to greater prosocial behaviour and fewer internalising and externalising problems, even after accounting for individual characteristics and selection into schools. We also expected that a more favourable relative to a less favourable (academic and socio-economic) school profile would be particularly beneficial for poor children.

3. To examine gender differences in the moderated (by school composition) effect of poverty on child behaviour.

We did not anticipate any gender differences in the (expected) moderator effect of school composition on child behaviour at this age.

We controlled for child and family/parent characteristics related to both poverty and child behaviour, including maternal psychological distress [32] and family structure. We also controlled for child cognitive ability and parental education, which, alongside family poverty, should account for families’ selective sorting into schools. Accounting for selection into schools is important if one is to ascertain whether school ‘effects’ are genuine or simply exist because individual pupil characteristics are not accounted for [38]. In our case, selection occurs if the sorting of pupils into schools is not independent from child behaviour, our outcome. For example, child cognitive ability at the beginning of school should be related to both internalising and externalising problems and selection into schools. Similarly, poorer or less educated families are more likely to have children who both attend lower-SES or lower-achieving schools and have more internalising and externalising problems. When estimating the effect of school academic composition (i.e., school-level academic achievement), we also controlled for the corresponding individual factor (i.e., the child’s own academic achievement). We did this to avoid committing the ecological fallacy, whereby inference occurs at the group level, but is actually attributable to confounding by individual factors [39]. When estimating the effect of school socio-economic composition (i.e., school-level free school-meal (FSM) eligibility), we did not control for the individual child’s FSM eligibility due to the strong correlation between family poverty and child FSM eligibility.

## Methods

### Participants and procedure

MCS (http://www.cls.ioe.ac.uk/mcs) is a longitudinal survey of 19,244 families drawing its sample from all births in the UK over a year, beginning on 1/9/2000. The MCS sample is disproportionately stratified to ensure adequate numbers in the four UK countries and electoral wards with disadvantaged or (in England) ethnic minority populations [40]. Ethical approval for MCS was gained from NHS Multi-Centre Ethics Committees, and parents gave informed consent before interviews took place. We used data from Sweeps 1–4, taking place when the children were around 9 months, and 3, 5 and 7 years, respectively. Our analytic sample (*n* = 7225) was derived as follows: using records for only one child per family (the first-born where there were twins or triplets), we started with 8,445 children who lived in England at Sweeps 3 (age 5, when most children in England start school full-time) and 4, as data on both school-level and individual achievement at primary school were only available to us for England. We then dropped those who were missing data on age 7 behaviour (*n* = 351), leaving us with 8094 children. Subsequently, we dropped those without information on what school they attended at Sweeps 3 and 4 (*n* = 214), resulting in a sample of 7880. MCS did not collect data on what schools children attended between sweeps (only what school they were attending at the time of the MCS interview). Therefore, we then excluded children who changed schools prior to Sweep 3 and/or changed schools more than once between Sweeps 3 and 4 (*n* = 282), leaving us with a sample of 7598. This meant that we selected only children attending up to two schools at ages 5–7, both of which had to be attended at the time of interview. School composition could only be available for state schools. Therefore, children attending fee-paying schools at the times of Sweeps 3 and 4 were excluded from the sample as well (*n* = 373). In our sample, children attended a total of 2948 schools at age 7, with a range of 1–26 MCS children attending the same school (at age 5, the total number of schools was 2749 and the range of MCS children attending the same school was 1–28). In all, 90 % of children in our sample did not change schools between ages 5 and 7. Hence, 10 % changed schools once.

#### Sample bias analysis and descriptives

Family poverty, school composition and child behaviour were significantly inter-related except for living sometimes in poverty and prosocial behaviour (Table 1). As expected, children in the analytic sample had more privileged backgrounds relative to children in the non-analytic sample.

**Table 1:** Correlations among the risk, moderator and outcome variables in the analytic sample

	1	2	3		4	5	6	7		8	9		10	11	12
Duration of poverty															
1. No. of sweeps in poverty	1														
2. Always poor (vs. never poor)	**0.66**	1													
3. Sometimes poor (vs. never poor)	**0.53**	**−0.27**	**1**												
Timing of poverty															
4. Poor at 9 months	**0.80**	**0.63**	**0.27**		**1**										
5. Poor at 3 years	**0.82**	**0.62**	**0.30**		**0.60**	1									
6. Poor at 5 years	**0.82**	**0.62**	**0.30**		**0.56**	**0.62**	1								
7. Poor at 7 years	**0.78**	**0.67**	**0.21**		**0.53**	**0.56**	**0.59**	1							
School factors (age 7)															
8. School KS1	**−0.36**	**−0.27**	**−0.15**		**−0.32**	**−0.31**	**−0.31**	**−0.30**		1					
9. School FSM	**0.49**	**0.36**	**0.20**		**0.42**	**0.41**	**0.41**	**0.40**		**−0.65**	1				
Child outcomes (age 7)															
10. Internalising	**0.23**	**0.17**	**0.10**		**0.19**	**0.21**	**0.21**	**0.20**		**−0.17**	**0.19**		1		
11. Externalising	**0.20**	**0.13**	**0.10**		**0.16**	**0.17**	**0.17**	**0.17**		**−0.14**	**0.16**		**0.39**	1	
12. Prosocial	**−0.07**	**−0.06**	−0.01		**−0.06**	**−0.07**	**−0.05**	**−0.07**		**0.06**	**−0.06**		**−0.21**	**−0.43**	1

Tests are two-tailed. All bolded coefficients are significant at *p* < 0.001. Pearson correlations were run when both variables had normal distributions and interval/ratio data. Spearman correlations were run when either or both variables had either a non-normal distribution or an ordinal measurement scale

*KS1* Key Stage 1 (scores), *FSM* free school meal (eligibility)

### Measures

School academic composition was measured with the school-average Key Stage^1^ 1 (KS1) scores (averaged across English, Maths and Science) of pupils in state-maintained schools collected during the January 2006 (corresponding with Sweep 3) and January 2009 (corresponding with Sweep 4) censuses, obtained from the School Data Unit at the Department for Education. The KS1 scores were banded into deciles based on all primary schools in England. KS1 scores are obtained at the end of year 2. Therefore, at age 5, these school-level scores apply to a different cohort of children from that of MCS. The individual MCS children’s academic achievement was measured with their KS1 average scores collected during the January 2009 census and obtained from the National Pupil Database. The individual KS1 scores were also banded into deciles. School socio-economic composition was measured with the percentage of pupils eligible for free school-meals (FSMs), also collected during the January 2006 and 2009 sweeps, banded into deciles based on all primary schools in England. All sensitive data were linked with MCS data in a secure environment using the unique reference number of each child’s school.

Child behaviour was operationalised, as explained, as internalising problems, externalising problems and prosocial behaviour, measured at age 7 with the parent-reported Strengths and Difficulties Questionnaire (SDQ) [41]. The SDQ is a 25-item scale measuring four difficulties (hyperactivity, emotional symptoms, conduct problems and peer problems) and prosocial behaviour. Item responses range 0–2. In line with recommended practice for community samples [42], the internalising problems scale comprised the 10 items from the emotional and peer problems subscales, and the externalising problems scale the 10 items from the hyperactivity and conduct problems subscales. Scores for each 10-item scale may range 0–20. The prosocial behaviour scale comprises five items and therefore has scores ranging 0–10. In our sample, internal consistency was at acceptable levels, and in line with other SDQ research [43]. Cronbach’s alpha values for age 7 outcomes were 0.80 (externalising problems), 0.71 (internalising problems) and 0.70 (prosocial behaviour).

Family poverty was measured with a binary indicator of whether the family income was below the poverty line, set as equivalised net family income at 60 % of the national median household income. We measured both the timing of poverty and its duration, as in Duncan, Brooks-Gunn and Klebanov [44]. To capture the timing of poverty, we estimated the effect of poverty separately for each age (9 months and 3, 5 and 7 years). Duration was measured in two ways:

1. Continuously: the number of sweeps the family was living below the poverty line, ranging 0–4.
2. Categorically: as a set of dummy variables where 1 = chronic poverty (living below the poverty line at all four sweeps), 2 = intermittent poverty (living below the poverty line at least once but not at every sweep), and 3 = never in poverty (not living below the poverty line at any sweep).

Key covariates were both parent/family-level and child-level. The family-level variables were maternal education (University degree or not by child’s age 7), maternal psychological distress (age 5), measured with the 6-item Kessler scale [45] and family structure (age 5; intact or not). The child-level variables were age in years (at Sweep 4, around age 7, when child behaviour was measured), gender, ethnicity and general intelligence (at the beginning of primary school at age 5). To measure general intelligence, regression factor scores were derived from principal components analysis of multiple age-adjusted ability assessment scores. Then the factor score was transformed into a standardized IQ score with a mean of 100 and a standard deviation of 15 [46]. At age 5, ability was assessed with the BAS Naming Vocabulary, BAS Pattern Construction (measuring spatial problem solving) and BAS Picture Similarities (measuring non-verbal reasoning) scales. All conditional models also accounted for the stratified sample design of MCS.

### Analytic plan

**Fig. 1:**
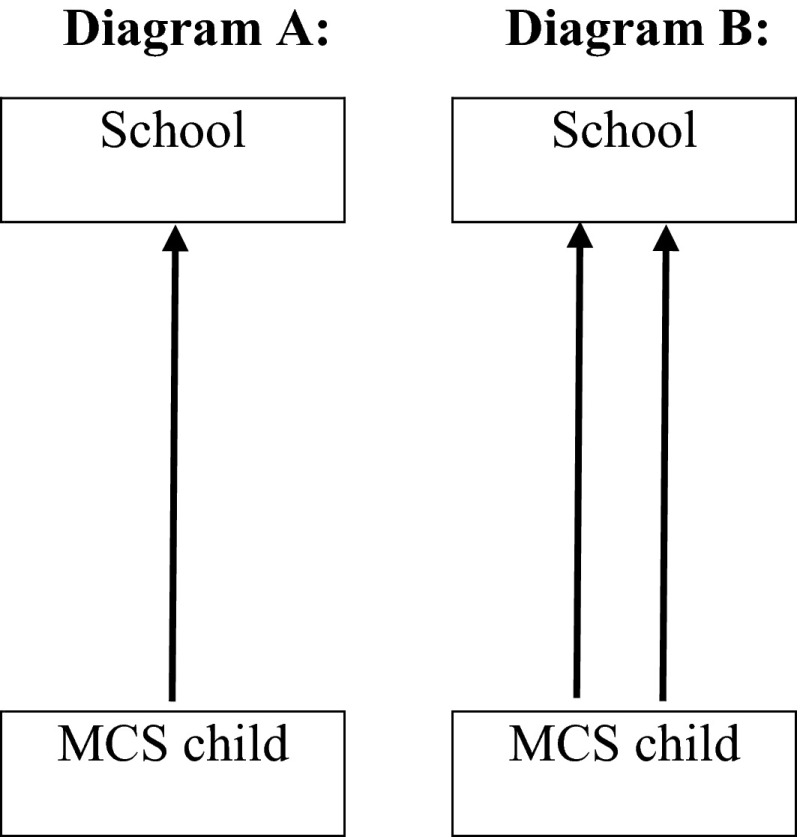
Classification diagrams for **a** a simple two-level nested model and **b** a two-level multiple membership model

We fitted two-level multiple membership models [47, 48]. Our models were two-level (child at level 1 and school at level 2) to avoid the underestimation of standard errors due to our hierarchical data structure where children were nested within schools. Schools where only one MCS child was in attendance (roughly half our sample) were included as they contribute to the estimates of individual-level characteristics in the fixed effects part of the model, even though they do not contribute to the variance between schools. Multiple membership models, where the lowest-level unit can be a member of more than one higher-level units (Fig. 1), are an extension to the standard multilevel framework. In our case, children (our lowest-level unit) can change school and therefore attend more than one school (higher-level unit) from ages 5 to 7. Each of these schools can, in theory, contribute to the child’s outcomes we considered. However, this may depend on the amount of time spent in each school which will vary depending on the child’s situation. Therefore, to account for the multiple membership in schools, the random school effect should be weighted by the length of time in the school. As MCS did not collect information on the amount of time children attended a given school, we assigned equal weights to the schools attended. Specifically, we assigned a weight of 50 % to each of the two schools if the child attended two schools, and a weight of 100 % to the school if the child attended only one school across ages 5 to 7. We also modelled our two school composition variables as weighted-averages across the schools children attended [49].

Our models were fitted using Markov chain Monte Carlo techniques in MLwiN. Therefore, we used the Bayesian deviance information criterion (DIC) [50], instead of the likelihood ratio test, to estimate the relative fit of the two-level multiple membership model, the simple two-level model and the single-level model. The DIC is a measure of the model’s overall fit and is penalised for the model’s parametric complexity.

We carried out a series of models (Table 2). In the unconditional model (Model 1), we examined the variation in child behaviour between schools. Model 2 added poverty, and Model 3 the child and parent/family covariates alongside the MCS design variables. Model 4 added the KS1 school variable and Model 5 the interaction between school-level KS1 scores and family poverty. Model 6 added to Model 3 the school variable measuring % FSM-eligible, and Model 7 the interaction between school-level FSM eligibility and poverty. As explained, when estimating the effect of school-average KS1 scores, we also controlled for the child’s own KS1 score. In Models 2–7, we examined both the timing and the duration of poverty. We ran each model for each of our three outcome variables. We also modelled both age 7 outcomes and, in separate models, age 7 outcomes controlling for outcomes at age 5 (to predict change in outcomes between ages 5 and 7).

**Table 2:** Model summary

Model	Variables
1 (unconditional)	Constant
2	Model 1 + Poverty
3	Model 2 + Covariates + MCS Design Strata
4	Model 3 + School KS1 + Child KS1
5	Model 4 + Poverty × School KS1
6	Model 3 + School FSM
7	Model 6 + Poverty × School FSM

Our dependent variables (indexing child behaviour) were externalising problems, internalising problems and prosocial behaviour at age 7. Three sets of conditional models were carried out to explore the roles of duration and timing of poverty: (1) duration treated as the number of sweeps in poverty, (2) duration measured categorically (always in poverty, sometimes in poverty and never in poverty) and (3) timing measured as being in poverty or not at ages 9 months, 3, 5 and 7 years. We also ran these conditional models controlling for age 5 child behaviour to predict changes in behaviour between ages 5 and 7. To test for any regional effects on child behaviour, we ran all conditional models controlling for region. Our findings remained the same (and region was not significantly related to any outcome at age 7). Also, neither school FSM nor school KS1 effects depended on region

*KS1* Key Stage 1 (scores), *FSM* free school meal (eligibility)

## Results

### Model 1

Accounting for the multiple membership of children in schools improved model fit compared to fitting either a simple two-level model or a single-level model (the DIC was lower for the multiple membership model). We then calculated the variance partition coefficient or the proportion of observed response variation that lies at each level of model hierarchy. All school random effects were significant although small. For internalising problems, for a child attending one school only, the random school effect was 6.2 % and the random child effect 93.8 %. Therefore, school contributed 6.2 % (intraclass correlation) of the variance in internalising problem scores. For a child attending two schools, the random school and child effects were 3.2 and 96.8 %. Therefore, the two schools as a whole contributed to only 3.2 % of the variance in scores, less than the single school among the non-movers, as expected. For externalising problems, if attending one school, the child effect was 95.9 % and the school effect 4.1 % (if attending two schools, the between-child and between-school variance was 97.8 and 2.2 %, respectively). For prosocial behaviour, if attending one school, the between-child variance was 97.8 % and the between-school variance was 2.2 % (if attending two schools, the numbers were 98.9 and 1.1 %, respectively). Therefore, there was evidence for a small amount of clustering within schools, particularly for internalising and externalising problems. However, we felt it was appropriate to account for even this small amount of clustering to reduce the possibility of overestimating our school effects, especially since our main study objective was to tease out a school-level effect. Below we present the results of Models 2–7 predicting both age 7 outcomes and change in outcomes between ages 5 and 7.

### Models 2–7: predicting age 7 outcomes

The cumulative effect of family poverty (i.e., the number of sweeps living below the poverty line) was significantly related to all three outcomes (Model 2), and was robust to family and child controls and the MCS design variables (Model 3, Table 3). The random effect of school remained significant, although it was reduced in size, after accounting for poverty, family and child controls and the MCS design variables. Although school-level KS1 scores were associated negatively with individual children’s externalising and internalising problems (but were unrelated to prosocial behaviour) prior to accounting for individual KS1 scores, the main effects of school KS1 scores were not significant on any of our outcomes after controlling for individual KS1 scores in Model 4 (Table 3). This suggests that the effect of school academic composition on children’s internalising and externalising problems was driven by the clustering of children into schools according to their academic performance. Furthermore, school KS1 scores did not interact with the number of sweeps in poverty to affect any child outcomes (Model 5). In Model 6 (Table 3), the main effect of school-level FSM eligibility was significant (and positive) for externalising and internalising problems. As with school-level KS1 scores, school-level FSM eligibility did not interact with cumulative poverty (Model 7). All random effects remained significant.

**Table 3:** Fixed effects estimates (poverty measured cumulatively) and variance covariance estimates for Models 3, 4 and 6 predicting internalising problems, externalising problems and prosocial behaviour

	Model 3			Model 4			Model 6		
	Externalising	Internalising	Prosocial	Externalising	Internalising	Prosocial	Externalising	Internalising	Prosocial
Fixed effects									
Constant	15.44* (1.44)	7.65* (1.13)	6.34* (0.64)	8.56* (1.55)	4.41* (1.25)	6.63* (0.77)	15.09* (1.47)	7.50* (1.16)	6.13* (0.71)
No. of sweeps in poverty	0.18* (0.04)	0.20* (0.03)	−0.05* (0.02)	0.12* (0.04)	0.16* (0.03)	−0.05* (0.02)	0.16* (0.04)	0.18* (0.03)	−0.05* (0.02)
MCS strata (Ref. = E-advantaged)									
E-disadvantaged	0.22* (0.10)	0.11 (0.08)	0.03 (0.05)	0.18 (0.11)	0.13 (0.09)	0.08 (0.05)	0.09 (0.11)	0.06 (0.09)	0.04 (0.05)
E-ethnic	0.50* (0.19)	0.25* (0.15)	−0.06 (0.09)	0.47* (0.21)	0.25 (0.17)	−0.01 (0.10)	0.41* (0.20)	0.12 (0.16)	−0.09 (0.09)
W-advantaged	0.11 (0.91)	−0.06 (0.72)	0.42 (0.44)	−0.08 (1.10)	−0.48 (0.89)	0.10 (0.56)	0.32 (0.92)	0.05 (0.72)	0.37 (0.45)
W-disadvantaged	0.28 (0.62)	0.15 (0.49)	0.19 (0.32)	0.60 (0.66)	0.36 (0.53)	0.29 (0.33)	0.47 (0.67)	0.06 (0.52)	0.22 (0.32)
S-advantaged	1.15 (0.93)	0.24 (0.73)	−0.20 (0.45)	1.64 (1.00)	0.14 (0.81)	−0.48 (0.50)	1.33 (0.94)	0.42 (0.74)	−0.09 (0.48)
S-disadvantaged	1.47 (1.42)	−0.91 (1.11)	−0.29 (0.68)	0.70 (1.75)	−0.47 (1.41)	0.15 (0.89)	1.40 (1.43)	−0.95 (1.12)	−0.29 (0.71)
NI-disadvantaged	1.64 (3.16)	5.07* (2.47)	−0.55 (1.55)	0.53 (3.08)	4.68 (2.48)	−0.27 (1.52)	1.84 (3.36)	5.23* (2.63)	−0.53 (1.57)
Age in years at Sweep 4	−0.87* (0.19)	−0.45* (0.15)	0.23* (0.08)	−0.11 (0.20)	−0.07 (0.16)	0.21* (0.10)	−0.86* (0.19)	−0.45* (0.15)	0.27* (0.09)
Girl	−0.97* (0.09)	−0.01 (0.07)	0.56* (0.04)	−0.84* (0.09)	0.07 (0.07)	0.54* (0.05)	−0.93* (0.09)	0.02 (0.07)	0.56* (0.04)
Ethnicity (Ref.: White)									
Mixed	−0.38 (0.24)	−0.17 (0.19)	0.04 (0.12)	−0.19 (0.26)	−0.09 (0.21)	−0.00 (0.13)	−0.43 (0.25)	−0.15 (0.20)	−0.004(0.12)
Indian	−0.68* (0.31)	0.24 (0.20)	0.03 (0.14)	−0.48 (0.31)	0.48 (0.25)	−0.09 (0.16)	−0.76* (0.30)	0.33 (0.24)	0.04 (0.14)
Pakistani/Bangladeshi	−0.93* (0.26)	0.01 (0.20)	0.08 (0.11)	−0.71* (0.28)	0.25 (0.23)	0.08 (0.15)	−0.95* (0.26)	0.05 (0.21)	0.07 (0.13)
Black/Black British	−1.13* (0.27)	−0.16 (0.21)	0.31* (0.13)	−1.20* (0.31)	−0.18 (0.25)	0.44* (0.16)	−1.35* (0.29)	−0.23 (0.23)	0.33* (0.13)
Other	−1.07* (0.44)	−0.27 (0.34)	0.53* (0.21)	−0.81 (0.48)	−0.20 (0.38)	0.41 (0.23)	−1.10* (0.48)	−0.15 (0.37)	0.51* (0.22)
Intelligence	−0.04* (0.00)	−0.02* (0.002)	0.00 (0.00)	−0.01* (0.004)	−0.01* (0.003)	0.001(0.002)	−0.04* (0.003)	−0.02* (0.003)	0.0038 (0.0017)
University-educated (M)	−0.67* (0.11)	−0.15 (0.09)	−0.04 (0.05)	−0.39* (0.13)	0.01 (0.11)	−0.09 (0.07)	−0.66* (0.12)	−0.11 (0.09)	−0.03 (0.06)
Psychological distress (M)	0.16* (0.01)	0.16* (0.01)	−0.03* (0.01)	0.14* (0.01)	0.16* (0.01)	−0.03* (0.01)	0.16* (0.01)	0.16* (0.01)	−0.03* (0.01)
Intact family	−0.67* (0.12)	−0.32* (0.10)	0.07 (0.06)	−0.53* (0.14)	−0.27* (0.11)	0.03 (0.07)	−0.70* (0.12)	−0.32* (0.10)	0.04 (0.06)
School KS1				0.03 (0.02)	−0.01 (0.02)	0.003(0.01)			
Child KS1				−0.33* (0.02)	−0.13* (0.02)	0.03* (0.01)			
School FSM							0.05* (0.02)	0.036* (0.016)	0.001 (0.01)
Random effects									
School-level	0.17* (0.08)	0.15* (0.07)	0.02* (0.004)	0.29* (0.09)	0.19* (0.07)	0.04* (0.01)	0.14* (0.04)	0.15* (0.04)	0.03* (0.01)
Child-level	10.35* (0.20)	6.32* (0.13)	2.37* (0.05)	9.45* (0.21)	6.10* (0.14)	2.36* (0.05)	10.35* (0.21)	6.34* (0.13)	2.38* (0.05)
DIC	29762.21	26958.83	21285.54	23466.77	21464.12	17047.84	27437.78	24888.85	19671.09

Northern Ireland-advantaged is missing because there were no children in our analytic sample who were born in a ward from this stratum category

*M* maternal, *E* England, *W* Wales, *S* Scotland, *NI* Northern Ireland, *KS1* Key Stage 1 (scores), *FSM* free school meal (eligibility), *DIC* Deviance Information Criterion

* *p* < 0.05 School-level variables are weighted averages of the values at Sweeps 3 and 4

We then modelled the duration of poverty categorically. In Model 2, the effects of chronic poverty (being poor in every sweep) and intermittent poverty (being poor in at least one but not every sweep) relative to never being poor were significant on all three outcomes. These effects remained significant in Model 3, with one exception. The effect of intermittent poverty was no longer significantly related to prosocial behaviour. As when modelling cumulative poverty, in Models 4 and 6 the effects of school-level KS1 scores were null, and school-level FSM eligibility was positively associated with externalising and internalising problems. Again, neither school-level variable moderated the effect of either chronic or intermittent poverty across childhood.

When measuring poverty in terms of timing, poverty at any age was associated with more externalising and internalising problems and less prosocial behaviour. These effects were partially attenuated but remained significant in Model 3. There were no significant interactions between school-level KS1 scores or school-level FSM eligibility and family poverty at any age (Models 5 and 7).

In the fully-adjusted model (Model 3), when measuring either the duration (continuously or categorically) or the timing of poverty, girls had fewer externalising problems and higher prosocial behaviour scores. There were several ethnic differences in our three outcomes. Relative to white children, Indian, black, Pakistani/Bangladeshi and ‘other ethnic’ children had fewer externalising problems. Black and ‘other ethnic’ children had higher prosocial behaviour scores than white children. General intelligence was related to all three outcomes. With regard to parent/family factors, mother’s education was related to fewer externalising problems, and intact family structure was associated with fewer externalising and internalising problems. Mother’s psychological distress predicted more externalising and internalising problems as well as less prosocial behaviour.

### Models 2–7: predicting change in outcomes from 5 to 7 years

As explained, we modelled change in child behaviour between ages 5 and 7 by controlling for age 5 child behaviour. In Models 2 and 3, all our poverty variables were related to changes in the outcomes. Cumulative poverty was related to an increase in internalising and externalising problems, and a decrease in prosocial behaviour. Chronic or intermittent poverty was related to an increase in externalising and internalising problems and to a decrease in prosocial behaviour. Poverty experienced at any of the three ages (9 months, 3 or 5 years) was associated with an increase in externalising and internalising problems. Additionally, poverty experienced at ages 3 or 5 was related to a decrease in prosocial behaviour. In Models 4 and 6, using any poverty measure, we found only one school main effect: higher school-level achievement predicted an increase in externalising problems, after controlling for individual achievement scores. We explored whether this newly-significant effect of school-level achievement (in the fully-adjusted model of externalising problems at age 7, the effect was also positive but not significant) occurred at parts or across the distribution of school-average academic performance. To test this, we categorised school-average academic performance into three groups:

1. High achievement (among the top three deciles of performance: deciles 8–10).
2. Medium achievement (among the middle four deciles of performance: deciles 4–7).
3. Low achievement (among the bottom three deciles of performance: deciles 1–3).

Our findings suggested a non-linear effect of school-level achievement on change in externalising problems during primary school. Compared to attending a low-achieving school (i.e., when attending a low-achieving school is the reference group), attending a high or medium-achieving school was associated with an increase in externalising problems from age 5 to 7. When attending a high or medium-achieving school is the reference group, attending a low-achieving school was related to a reduction in problems from age 5 to 7. There were no protective effects of either academic or socio-economic composition on any outcomes in models adjusting for child and family/parent covariates (Models 5 and 7).

### Gender differences

Finally, we examined whether school composition may moderate the effects of poverty (measured both categorically and continuously, and in terms of timing), differently for boys and girls, on age 7 child behaviour and on change in child behaviour between 5 and 7 years. As expected, there were no gender differences in the (null) moderator effects.

## Discussion

There is little research on the role of school composition in young children’s behaviour. This study sought to examine whether primary school composition has promotive or protective effects for parent-reported child behaviour in a large, representative sample of families in England. Our first aim was to model the relationship between family poverty and child behaviour (internalising and externalising problems and prosocial behaviour) at age 7. As expected, and in line with previous research [3], we found that the effects of poverty were strong and robust to adjustment for child and parent background characteristics, as well as school intake characteristics. Our second aim was to explore the role of school composition—academic and socio-economic—in both predicting child behaviour and moderating the effects of poverty on child behaviour. School composition (either socio-economic or academic) did not interact with either duration or timing of poverty to predict child outcomes. However, there was a weak main effect of school socio-economic composition on internalising and externalising problems at age 7, such that, irrespective of own poverty status, attending a school with a higher proportion of poor children was associated with more internalising and externalising problems. This effect remained significant even after controlling for individual and other family factors related to child behaviour and selection into schools, such as ethnicity, intelligence, maternal education, family structure and maternal psychological distress. As for the role of school academic composition in the child outcomes we considered, we did not find any effect at age 7 once accounting for individual academic performance, although it appeared that children in the lowest-achieving primary schools improved more in terms of externalising behaviour from ages 5 to 7. Whether this reflects a genuine school effect, different parental expectations of behaviour for those attending high versus low-performance educational institutions, or simply the fact that children in low-achieving schools start school with a higher level of externalising problems and therefore can easily improve after 2 years, is unclear. Having detailed school environment data (e.g., on school policies, school connectedness or perceptions of teacher support) would help testing the first hypothesis, and having observational or teacher-reported data on child behaviour would help testing the second. However, MCS did not collect such data (it did collect teacher-reported SDQ scores at the age 7 survey but the level of non-response was very high). Our last aim was to examine gender differences in the moderated (by school composition) effect of poverty on child behaviour. As expected, we found no gender differences. That is, the null ‘protective’ effects of academic or socio-economic school mix we identified did not differ for boys and girls.

A strength of our study is that we accounted for between-school variability in children’s outcomes. There was, however, a relatively small amount of variation between schools in child behaviour in our unadjusted models (6.2 and 4.1 % in internalising and externalising problems), reflecting previous research on the role of school in child mental health [24]. The between-school differences remained significant in all models, and hence were not fully absorbed by child and family characteristics or school composition. The small amount of between-school variation may be partially due to the design of MCS and therefore the (limited) extent of hierarchy in our data. Roughly half of the sample did not attend school with other MCS children. Thus, our ability to estimate and therefore understand school effects was limited without information on more of the individual pupils in the schools attended. Furthermore, as mentioned, we did not have measures of the school environment. There is certainly evidence that student perceptions of teacher support and school connectedness (or ‘school belonging’) are associated with better emotional health among individual students, at least in adolescence [51]. Therefore, future research should also explore contextual, rather than only compositional, measures of the school environment including whole-school policies, leadership and school climate (e.g., engagement), as well as aspects of the more proximal classroom environment (e.g., classroom composition, classroom management and teacher quality).

We did not find that favourable school composition characteristics were particularly beneficial for the behaviour of children from poor families. Children from poor families were a high-risk group for, particularly, internalising and externalising problems irrespective of the academic or socio-economic mix of the school they attended, and as such they should be prioritised in interventions to promote child mental health. However, our study also showed that attending a school with a privileged socio-economic intake (on the assumption, of course, that not being on free school meals is an acceptable approximation of privilege) was associated with fewer internalising and externalising symptoms (but not more prosocial behaviour). Thus, it appears that, as early as at the beginning of primary school in England, the grouping of high-SES pupils into a school creates conditions associated with even better emotional/behavioural outcomes than would be expected from individual pupils’ SES alone. As we have theorised, this may be due to positive peer influences demonstrating good behaviour. If this were true, then peer-based interventions in schools to improve children’s emotional and behavioural regulation would be a natural implication of this finding. Of course, this school ‘effect’ we found may be instead (or also) due to other school characteristics associated with social mix (e.g., greater parental involvement in learning, higher-quality teachers or superior managerial processes within schools [33]). In that case, interventions should be developed to target the improvement of these specific characteristics to promote pupils’ mental health. Future research should therefore first explore the mechanisms of this effect to determine the best intervention approach. The nonsignificant effect of school intake on prosocial behaviour echoes previous findings that empathy and consideration for others seems to be an individual difference driven more by family than extra-familial influences [52].

## Footnotes

1 Key stages are stages of the state education system in England. Key Stage 1 applies to ages 5–7 (years 1–2). Children are assessed in English, Maths and Science at the end of Key Stage 1.
